# A distinctively expressed long noncoding RNA, RP11‐466I1.1, may serve as a prognostic biomarker in hepatocellular carcinoma

**DOI:** 10.1002/cam4.1565

**Published:** 2018-05-23

**Authors:** Junyong Zhang, Di Zhang, Qi Zhao, Jianni Qi, Xiao Li, Chengyong Qin

**Affiliations:** ^1^ Department of Gastroenterology Shandong Provincial Hospital Affiliated to Shandong University Jinan, Shandong China; ^2^ Central Laboratory Shandong Provincial Hospital Affiliated to Shandong University Jinan, Shandong China

**Keywords:** diagnosis, hepatocellular carcinoma, lipid metabolism, long noncoding RNAs, prognosis

## Abstract

It is urgent to explore effective diagnostic and prognostic biomarkers for hepatocellular carcinoma (HCC). Now, both lncRNAs and lipid metabolism are involved in tumor pathogenesis. Long noncoding RNA, RP11‐466I1.1, could likely be linked to lipid metabolism according to our bioinformatics analysis, yet studies about RP11‐466I1.1 expression in tumors and its potential functions are still lacking. We aimed to explore the expression and correlations with clinical features of a long noncoding RNA, RP11‐466I1.1, and further analyze its diagnostic and prognostic values in hepatocellular carcinoma. Expression levels of RP11‐466I1.1 were detected by quantitative real‐time PCR (qRT‐PCR) in tissue and serum level, and expression differences were analyzed by independent 2‐tailed *t* tests. Clinical features were obtained, and their correlations with RP11‐466I1.1 were analyzed by chi‐squared test. Receiver operating characteristic (ROC) curve was performed to assess the diagnostic value. Kaplan‐Meier method and log‐rank test were used to evaluate the prognostic value of RP11‐466I1.1. Results showed that RP11‐466I1.1 was upregulated in HCC tissues (*P* < .01) and serum (*P* < .05). Significant upregulation of RP11‐466I1.1 in HCC tissues with poor histological grade (*P* < .01) and incomplete tumor capsule (*P* < .01) was found compared to that with better histological grade and complete tumor capsule, respectively. The diagnostic value of RP11‐466I1.1 was not supported by ROC curve analysis (AUROC=0.665, *P* = .079). Yet, the significant correlation of RP11‐466I1.1 with poor prognosis indicated its potential prognostic value in HCC. This study suggested that RP11‐466I1.1 is distinctively expressed in HCC and may serve as a promising novel prognostic biomarker. The concrete mechanisms of RP11‐466I1.1 playing roles in HCC pathogenesis need further study.

## 1 INTRODUCTION

Hepatocellular carcinoma (HCC), accounting for 70%‐90% of primary liver cancer,^1^ is the third leading cause of cancer‐related death worldwide.^2^ The insufficiency of early diagnosis and the followed poor prognosis, which is usually associated with high metastasis rate and recurrence after nonradical treatment due to being detected at an advanced stage, are responsible for its high mortality mostly.^3^ For the purpose of improving the early diagnosis of HCC, several biomarkers besides α‐fetoprotein (AFP) have been investigated, such as des‐gamma‐carboxyprothrombin (DCP), osteopontin, α‐fucosidase, glypican‐3, and Dickkopf‐1 (DKK1), all of which however showed clear limitations.^4^, ^5^ Therefore, it is an urgent need for better management of HCC to explore effective diagnostic and prognostic biomarkers.

Long noncoding RNAs (lncRNAs), a class of noncoding transcripts with the length of more than 200 nucleotides, have been recognized as functional fragments in many biological processes by targeting related genes.^6^ So far, several HCC‐related lncRNAs, such as HULC, HOTAIR, and MALAT1, have been identified, whose dysregulation can responsible for HCC pathogenesis partially through regulating cell proliferation and apoptosis, angiogenesis, and tumor metastasis.^7^ On the other hand, lncRNAs are studied widely as key regulators of lipid metabolism. Thirty lncRNAs enriched in major lipid metabolism‐related tissue, including liver, muscle, or adipose tissues, are identified which may play crucial roles in lipid metabolism.^8^ For example, lncLSTR has been indicated regulating plasma triglyceride (TG) levels through a TDP‐43/FXR/apoC2‐dependent pathway.^9^

In this study, we focus on a long noncoding RNA, RP11‐466I1.1, which was firstly identified as an upregulated lncRNA in response to lipopolysaccharide (LPS) stimulation in human umbilical vein endothelial cells (HUVECs) by Singh et al^10^. The transcript of RP11‐466I1.1 is encoded by a non‐protein‐coding sequence located on human chromosome 11 with the length of 492bps. The article from Singh et al is currently the first and the only one publication involving RP11‐466I1.1. And there is no study to date involving its any functions in some biological process or revealing whether it plays roles in some tumor pathogenesis, such as HCC.

For learning more, bioinformatics analysis of RP11‐466I1.1 was carried out. At first, we evaluated the protein‐coding potential of RP11‐466I1.1 using Coding Potential Calculator (CPC) and Coding Potential Assessment Tool (CPAT), 2 widely used algorithms.^9^ According to CPC scores, the transcripts are classified as “coding” (score > 0) and “noncoding” (score < 0).^11^ And the CPC score of lncRNA RP11‐466I1.1 was −0.740916, indicating a noncoding RNA. For CPAT, with a cutoff value of 0.36, the transcripts are classified as “coding” when score is more than 0.36 and “noncoding” when <0.36.^12^ After calculation, RP11‐466I1.1 was also identified as a noncoding lncRNA with a CPAT score of 0.04. Then, we analyzed the sequence of RP11‐466I1.1 by NCBI database blast (basic local alignment search tool), with the discovery of several homologous genes which was related to lipid metabolism, for example, lipoprotein lipase (LPL) gene and PPAR‐gamma gene, particularly. Homology analysis demonstrated that 55% sequence of RP11‐466I1.1 was homologous to LPL gene, sharing a high sequence identity of 85%. Similarly, the homologous sequence to PPAR‐gamma accounts for 58% sequence of RP11‐466I1.1, with an identity up to 89%. Although it was not the proof enough, the data resulting from bioinformatics analysis strongly reminded us of the possible correlation between RP11‐466I1.1 and lipid metabolism.

Lipid dysmetabolism has been extensively illustrated in cancers^13^, ^14^, ^15^ and been considered as a vital contributor to tumor pathogenesis with possible mechanisms being researched in many human cancers.^16^, ^17^, ^18^, ^19^ What is more, a recent publication from Lee et al^20^ indicated that the dysregulated expressions of lipid metabolism‐related signaling hub genes may underlie HCC pathogenesis. The above descriptive analysis of the potential correlations among RP11‐466I1.1, lipid metabolism, and HCC suggested us a bold assumption that RP11‐466I1.1 may be involved in HCC pathogenesis through regulating lipid metabolism.

Although evidence is far from adequate, this assumption is still worthy of study and there is a long way to go to validate it. First of all, before exploration of the roles and mechanisms, the expression pattern of RP11‐466I1.1 in cancers must be figured out. Therefore, in this study, we aimed to detect the expression levels of RP11‐466I1.1 in human HCC tissues and serums, analyze its correlations with clinical features, and further investigate its diagnostic and prognostic values of HCC.

## 2 MATERIALS AND METHODS

### 2.1 Patients and clinical specimens

A total of 83 patients, including 72 patients with HBV‐related HCC and 11 hepatic hemangioma patients (negative controls), who underwent surgery, were recruited in this study between 2012 and 2013 at the Shandong Provincial Hospital Affiliated to Shandong University.^21^ HBV infection was diagnosed by clinical laboratory tests, and the final diagnosis of HCC was determined by pathological results. No patients had received chemotherapy, radiotherapy, or biotherapy before surgery. Three specimens were collected from each patient with HBV‐related HCC including HCC tissue, matched distal noncancerous liver tissue, and 5 mL venous peripheral blood before surgery. And liver tissue and 5 mL venous peripheral blood were obtained from each negative control patient as well. All patients were informed consent. This study was approved by the Medical Institutional Ethical Committee of the Provincial Hospital Affiliated to Shandong University and performed in accordance with the ethical standards as laid down in the 1964 Declaration of Helsinki and its later amendments or comparable ethical standards.

### 2.2 Clinical features

Clinical features of all patients were available and collected as shown in Table 1. The histopathological data including tumor size, cirrhosis, histological grade, metastasis, and tumor capsule of each tissue specimen were obtained from pathological report. The metastasis was defined as the movement of cancer cells to lymph node or other parts of the body. Laboratory parameters including AFP, carcinoembryonic (CEA), and γ‐glutamyltransferase (γ‐GT) were determined quantitatively by routine methods.^21^ Information about Child‐Pugh classification and BCLC staging were also collected. All processes were performed according to relevant instructions.

**Table 1:** Correlations between lncRNA RP11‐466I1.1 expression and the clinicopathological variables of patients with HCC

Characteristic	No. of patients (N = 72)	lncRNA RP11‐466I1.1 expression		*P* value
		Low	High	
Age (y)				
<50	29	16	13	.4710
≥50	43	20	23	
Sex				
Male	48	23	25	.6171
Female	24	13	11	
Tumor size				
<5 cm	28	11	17	.1469
≥5 cm	44	25	19	
Cirrhosis				
Positive	38	15	23	.0590
Negative	34	21	13	
Histological grade				
Good	14	12	2	.0049**
Moderate	43	20	23	
Poor	15	4	11	
Metastasis				
With	18	6	12	.1025
Without	54	30	24	
Tumor capsule				
Complete	26	22	14	.0055**
Incomplete	46	14	32	
AFP (ng/mL)				
≤20	40	24	16	.0578
>20	32	12	20	
CEA (ng/mL)				
≤10	58	30	28	.5515
>10	14	6	8	
γ‐GT (U/L)				
≤40	20	12	8	.2926
>40	52	24	28	
Child‐Pugh classification				
A	68	35	32	.3570
B	5	1	4	
C	0	0	0	
BCLC staging				
0	11	8	3	.1901
A	61	28	33	
B/C/D	0	0	0	

AFP, α‐fetoprotein; CEA, carcinoembryonic antigen; HCC, hepatocellular carcinoma; γ‐GT, γ‐glutamyl transpeptidase.

For the expression of lncRNA RP11‐466I1.1, median expression level was used as the cutoff. Data were analyzed by chi‐squared test. The difference was statistically significant when *P* < .01 (**) or *P* < .05 (*).

### 2.3 RNA extraction, reverse transcription, and quantitative real‐time PCR (qRT‐PCR)

Total RNA was extracted from both tissue specimens and serum specimens using the TRIzol reagent (Invitrogen, USA). The PrimeScript RT Reagent Kit (Takara Bio, China) was used for reverse transcription of lncRNA. Real‐time quantitative PCR was performed using the SYBR Premix Ex Taq (Takara Bio, China).^21^ The manufacturer’s instructions of each kit were followed during the whole process. GAPDH was used as an internal reference for normalization of lncRNA. Specific primers for RP11‐466I1.1 and GAPDH were designed as shown in Table 2.

**Table 2:** Specific primers used for qRT‐PCR

Name		Sequence (5′→3′)
GAPDH	Forward	CCAGGGCTGCTTTTAACTCT
	Reverse	GGACTCCACGACGTACTCA
RP11‐466I1.1‐a	Forward	CACCAAAGCAACAAGATGTGGA
	Reverse	AGGCTGGCCAAATTAGTCTGA
RP11‐466I1.1‐b	Forward	ACTAATTTGGCCAGCCTCTCC
	Reverse	CCAAACCCATCTGCTAGTGT
RP11‐466I1.1‐c	Forward	TAATTTGGCCAGCCTCTCCG
	Reverse	TTTGCTTGCCCAAAGGATTGC

RP11‐466I1.1‐a, primer for target fragment a of RP11‐466I1.1; RP11‐466I1.1‐b, primer for target fragment b of RP11‐466I1.1; RP11‐466I1.1‐c, primer for target fragment c of RP11‐466I1.1.

### 2.4 Statistical analysis

All of the data were analyzed using SPSS 16.0 statistical software (SPSS Inc., Chicago, IL, USA) and displayed using GraphPad Prism software (GraphPad Software, San Diego, CA, USA). The expression level of lncRNA in the specimen was calculated using the 2‐∆CT method relative to GAPDH. Chi‐squared test was used to evaluate the relationship between lncRNA RP11‐466I1.1 and clinical features, and independent 2‐tailed *t* test was used to analyze the statistical differences in RP11‐466I1.1 expression in different groups. To evaluate the diagnostic value of RP11‐466I1.1 in HCC, receiver operating characteristic (ROC) curve was performed and the area under the ROC curve (AUROC) was calculated. Kaplan‐Meier method and log‐rank test were used to evaluate the prognostic value of RP11‐466I1.1 in HCC. The difference was statistically significant when *P* < .01 (**) or *P* < .05 (*).

## 3 RESULTS

### 3.1 RP11‐466I1.1 was upregulated in HCC tissue specimens

**Figure 1:**
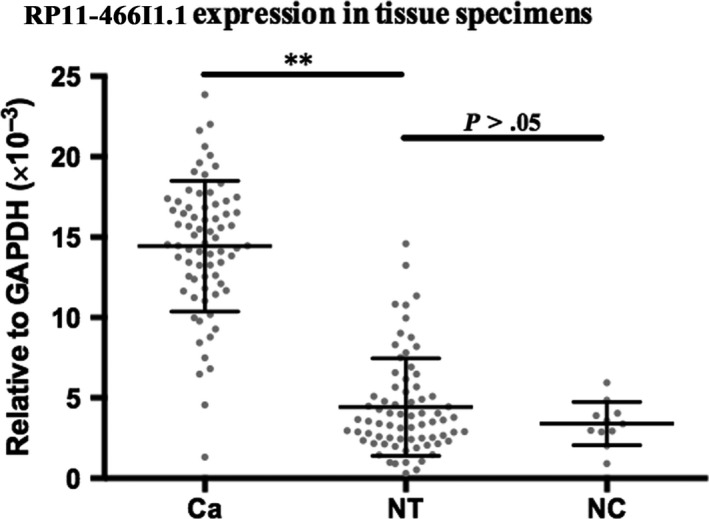
Upregulation of RP11‐466I1.1 in hepatocellular carcinoma (HCC) tissues. The expression of lncRNA in the specimen was calculated using the 2‐∆CT method relative to GAPDH. Differences between groups were analyzed by independent 2‐tailed *t* test. Result showed that expression level of RP11‐466I1.1 was significantly upregulated in HCC tissues compared with that of matched distal noncancerous liver tissues (*P* < .01**), while no significant difference was found between distal noncancerous liver tissues and normal liver tissues (*P* > .05)

The RP11‐466I1.1 expression levels in 72 HCC tissues, 72 matched distal noncancerous liver tissues, and 11 normal liver tissues (negative controls) were detected by qRT‐PCR, respectively. Results showed that, compared to matched distal noncancerous liver tissues, the expression level of RP11‐466I1.1 was significantly upregulated in HCC tissue specimens (*P* < .01**, Figure 1), while no significant difference was found between distal noncancerous liver tissues and normal liver tissues (*P* > .05, Figure 1). These results suggested that RP11‐466I1.1 may play vital roles in HCC pathogenesis.

### 3.2 Correlation between RP11‐466I1.1 and clinical features of HCC

The correlations between lncRNA RP11‐466I1.1 expression and the clinicopathological variables of patients with HCC were assessed as shown in Table 1. According to results, there was no significant correlation between RP11‐466I1.1 and clinical features including age, sex, tumor size, cirrhosis, metastasis, AFP, CEA, and γ‐GT, Child‐Pugh classification, and BCLC staging. However, RP11‐466I1.1 expression level was significantly correlated with the other 2 clinical features, histological grade and tumor capsule, described as follows.

#### 3.2.1 Histological grade (deterioration severity)

**Figure 2:**
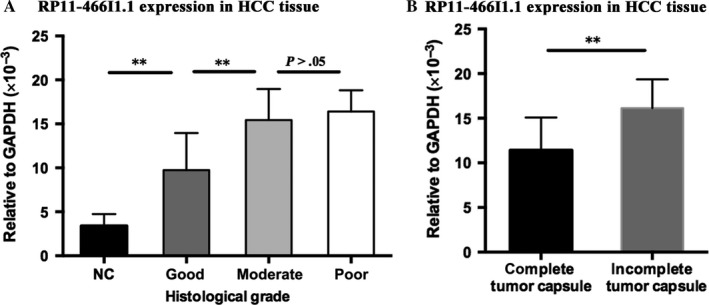
Significant correlations of RP11‐466I1.1 with histological grade and tumor capsule. The expression of lncRNA in the specimen was calculated using the 2‐∆CT method relative to GAPDH. Differences between groups were analyzed by independent 2‐tailed *t* test. A, Compared to that with normal tissue, RP11‐466I1.1 was significantly upregulated in hepatocellular carcinoma (HCC) tissues with good histological grade (*P* < .01**). Furthermore, RP11‐466I1.1 was significantly upregulated in HCC tissues with moderate histological grade compared to that with good histological grade (*P* < .01**). However, no significant difference was found between HCC tissues with moderate and poor histological grade (*P* > .05). B, There was significantly upregulation of RP11‐466I1.1 expression in HCC tissues with incomplete capsule compared to that with complete capsule (*P* < .01)

Seventy‐two HCC tissues specimens were divided into good, moderate, and poor grades according to histological differentiating degree. Relationships of RP11‐466I1.1 expression levels among 11 normal tissues, 14 good grade HCC tissues, 43 moderate grade HCC tissues, and 15 poor grade HCC tissues were analyzed. Compared with normal tissue, RP11‐466I1.1 expression was significantly upregulated in HCC tissues with good histological grade (*P* < .01**, Figure 2A). Furthermore, RP11‐466I1.1 expression was significantly upregulated in HCC tissues with moderate histological grade compared to that with good histological grade (*P* < .01**, Figure 2A). However, no significant difference was found between HCC tissues with moderate and poor grade tissues (*P* > .05, Figure 2A). The significant correlation of RP11‐466I1.1 with the differentiated degree of HCC tissues indicated potential roles RP11‐466I1.1 may play during the abnormal proliferation of HCC cells and a possible correlation between the high expression of RP11‐466I1.1 and poor prognosis of HCC. However, the slight difference of RP11‐466I1.1 expression between HCC tissues with moderate and poor grade cannot support this finding, which need further study.

#### 3.2.2 Tumor capsule (invasion ability)

According to the tumor capsule integrity, 72 HCC tissues were separated into 2 groups, 46 HCC tissues with complete capsule and 26 HCC tissues with incomplete capsule. After comparative analysis, there was significant upregulation of RP11‐466I1.1 in HCC with incomplete capsule compared to that with complete capsule (*P* < .01, Figure 2B). This result indicated that the high expression of RP11‐466I1.1 may be closely associated with the poor prognosis of HCC through facilitating tumor invasion.

### 3.3 Stability of RP11‐466I1.1 in serum

**Figure 3:**
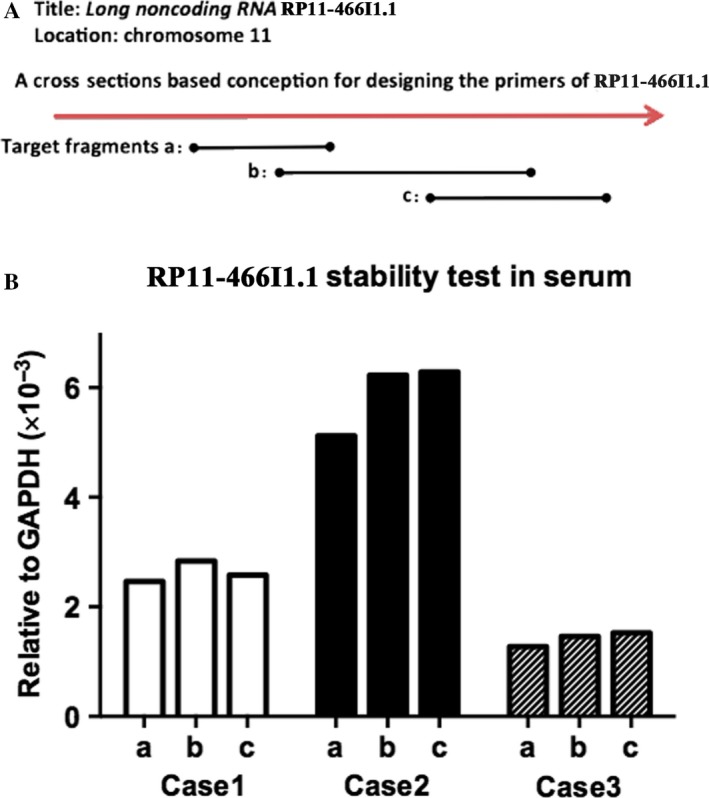
Stability of RP11‐466I1.1 expression in serum. The expression of lncRNA in the specimen was calculated using the 2‐∆CT method relative to GAPDH. A, Three specific primers (a,b,c) were designed whose corresponding target fragments crossed different connection segments. B, Three kinds of target fragments were transcribed in each tissue specimen using the 3 specific primers, whose concentration levels were pretty consistent in the same specimen

Considering that it was a connectome of several nucleotide fragments transcribed from DNA, RP11‐466I1.1 may be cut by RNase in serum. So in order to validate the stability of RP11‐466I1.1 in serum, 3 specific primers were designed whose corresponding target fragments crossed different connection segments as shown in Figure 3A. We detected the concentration level of 3 kinds of target fragments in each tissue specimen, respectively, and analyzed their differences. Results showed that the concentration levels of the 3 target fragments in the same specimen were pretty consistent (Figure 3B), suggesting good stability of RP11‐466I1.1 in serum. To rule out the possible effects of circular DNA, control experiments were performed. For control group, the total RNA extracted by TRIzol reagent (Invitrogen, USA) but not treated with PrimeScript RT Reagent Kit (TaKaRa, China) for reverse transcription. Then real‐time quantitative PCR was performed using the SYBR Premix Ex Taq (Takara Bio, China) under the same experimental conditions as the experimental group. That is, 480 PCR system (Roche) was used, and the temperature cycle consisted of the following: 95°C for 1 minute followed by 45 cycles of 95°C for 5 seconds, 65°C for 30 seconds, followed by 72°C for 30 seconds. The results showed no corresponding products were amplified. Therefore, the RP11‐466I1.1 expression level detected in serum in the experimental group was accurate.

### 3.4 RP11‐466I1.1 was upregulated in HCC serum

**Figure 4:**
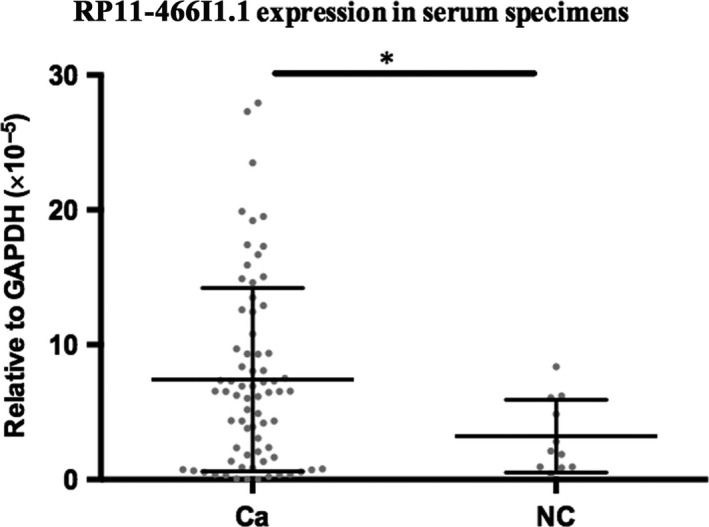
RP11‐466I1.1 was upregulated in hepatocellular carcinoma (HCC) serum. The expression of lncRNA in the specimen was calculated using the comparison 2‐∆CT method relative to GAPDH. Differences between groups were analyzed by independent 2‐tailed *t* test. In comparison with that in negative control serum, an obvious upregulation of RP11‐466I1.1 in HCC serum was found (*P* < .05*)

The RP11‐466I1.1 expression levels in 72 HCC serum collected before surgery and 11 negative control sera were detected by qRT‐PCR, respectively. In comparison with that in negative control serum, an obvious upregulation of RP11‐466I1.1 in HCC serum was found (*P* < .05*, Figure 4). Control experiments without treated with PrimeScript RT Reagent Kit for reverse transcription were also carried out to rule out the effects of circular DNA. As it was stable in serum and significant upregulation was demonstrated in HCC serum, RP11‐466I1.1 may be considered as a novel biomarker in the serum of patients with HCC.

### 3.5 Exploration of the diagnostic and prognostic values of RP11‐466I1.1 in HCC

#### 3.5.1 Evaluation of the diagnostic value

**Figure 5:**
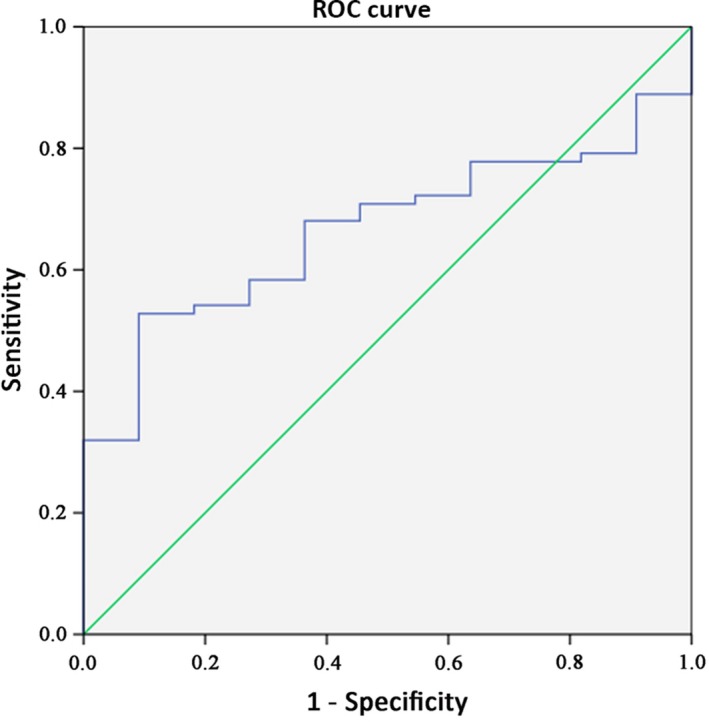
Exploration of the diagnostic value of RP11‐466I1.1 in hepatocellular carcinoma (HCC). The expression of lncRNA in the specimen was calculated using the comparison 2‐∆CT method relative to GAPDH. Receiver operating characteristic (ROC) curve was performed, and area under the ROC curve (AUROC) was calculated to evaluate the diagnostic value of RP11‐466I1.1 in HCC. The AUROC was 0.665 and *P*‐value was .079 (*P* > .05), indicating that RP11‐466I1.1 cannot serve as an effective diagnostic biomarker in HCC

ROC curve was performed to evaluate the diagnostic value of RP11‐466I1.1 in HCC. An AUROC of 0.665 with a *P*‐value of .079 (*P* > .05, Figure 5) indicated that RP11‐466I1.1 cannot serve as an effective diagnostic biomarker in HCC.

#### 3.5.2 Evaluation of the prognostic value

**Figure 6:**
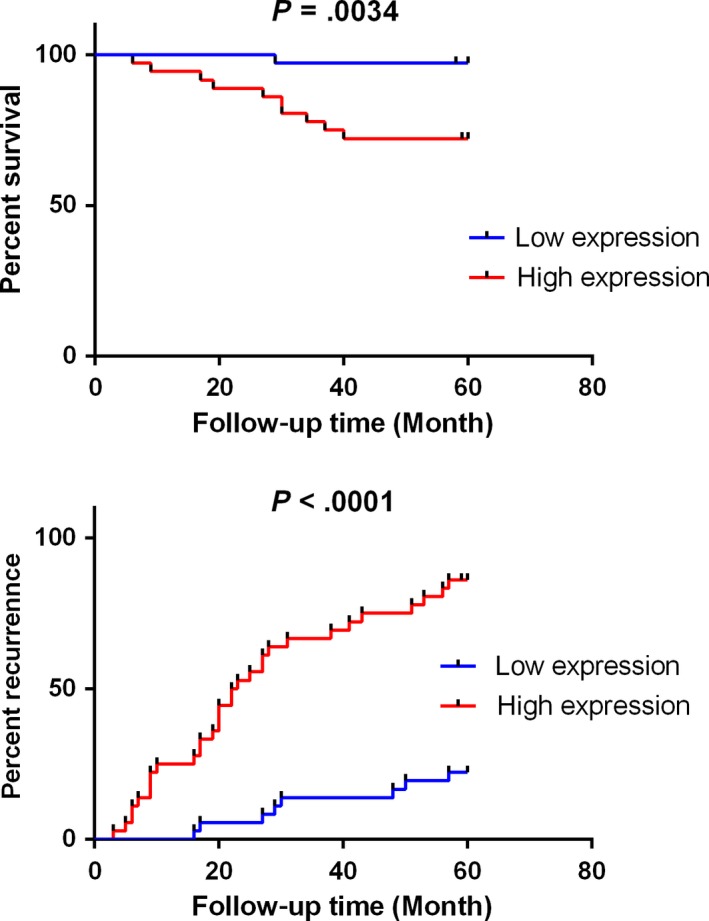
Exploration of the prognostic value of RP11‐466I1.1 in hepatocellular carcinoma (HCC). The expression of lncRNA in the specimen was calculated using the comparison 2‐∆CT method relative to GAPDH. The median expression level of lncLMA in HCC serum was used as the cutoff. Kaplan‐Meier analysis and log‐rank test were performed to evaluate the correlation between RP11‐466I1.1 expression and HCC survival and recurrence rate. High expression of RP11‐466I1.1 was correlated with lower survival rate (*P* = .0034) and higher recurrence rate (*P* < .0001)

For the purpose of prognosis assessment, Kaplan‐Meier method and log‐rank test were performed. As is shown in Figure 6, high expression of RP11‐466I1.1 was correlated with lower survival rate (*P* = .0034) and higher recurrence rate (*P* < .0001), indicating a significant correlation between the high expression of RP11‐466I1.1 and poor prognosis of HCC. This result suggested that RP11‐466I1.1 may serve as a promising prognostic biomarker in HCC.

## 4 DISCUSSION

Hepatocellular carcinoma is a highly prevalent tumor worldwide and threatens human health seriously, which is characterized by insidious onset, high malignancy, frequent metastasis and recurrence and poor prognosis. Although treatment of HCC has improved greatly in recent decades, radical resection on the basis of early diagnosis is still the main chance to achieve long‐term cancer‐free survival.^22^ At present, early diagnosis of HCC mainly relies on US, AFP, or combination of both.^22^ Due to their limitations, lots of researches have focused on exploring more effective biomarkers to improve the early diagnosis of HCC.^4^

LncRNAs, since been recognized as functional fragments in biological and pathological processes, have been demonstrated participating in HCC pathogenesis.^6^, ^7^ On the other hand, lncRNAs have also been identified as key regulators in lipid metabolism,^8^, ^9^ whose deregulation participating in HCC pathogenesis has been widely studied as a pointcut of searching for diagnosis and prognosis indicators. Taken together, these discoveries strongly prompt us to explore the relationships among lncRNAs, lipid metabolism deregulation, and HCC pathogenesis.

Long noncoding RNA, RP11‐466I1.1 is a newly discovered lncRNA reported by Singh et al^10^ firstly. Our bioinformatics analysis suggested the potential relationship between RP11‐466I1.1 and lipid metabolism for the high homology with lipid metabolism‐related genes including LPL and PPAR‐gamma. Yet, further studies about RP11‐466I1.1 expression trend, roles, or mechanisms in cancers are still lacking.

In the present study, we found that RP11‐466I1.1 was significantly upregulated in HCC tissues (*P* < .01) compared to that in matched distal noncancerous liver tissues. When we further investigated the correlation between RP11‐466I1.1 and HCC, we found significant upregulation of RP11‐466I1.1 in HCC tissues with poorer histological grade (*P* < .01) and incomplete tumor capsule (*P* < .01) compared to that with better histological grade and complete tumor capsule, respectively. These results together indicated that RP11‐466I1.1 may be an important participant of HCC pathogenesis by affecting HCC cells proliferation or facilitating tumor invasion with unknown mechanisms. Before further study to explore the concrete mechanisms, RP11‐466I1.1 may be firstly applied to clinical diagnosis or prognosis as effective biomarkers. In our following study, we found a significant upregulation in HCC serums (*P* < .05) compared with that in negative controls, which made it possible to be used as a noninvasive diagnostic or prognostic marker. However, its diagnostic value was not supported by the ROC curve analysis (AUROC = 0.665, *P* = .079). Prognostic value analysis revealed significant correlation between high expression of RP11‐466I1.1 and poor prognosis of HCC, which means RP11‐466I1.1 can be considered as a promising prognostic biomarker of HCC.

Several problems or limitations need to be considered in our study. First, in our study only 11 hepatic hemangioma patients were included as negative controls, which is far less than 72 patients with HCC. Adding some negative control samples would be better. However, hepatic hemangioma is a kind of benign disease which can be diagnosed by imaging methods and does not need surgical treatment generally, in which case, it is really hard to collect more NC samples which can provide liver tissue and serum simultaneously. Second, besides the histological grade and tumor capsule, no significant correlation was found between RP11‐466I1.1 expression level and other prognosis‐related indicators, especially including tumor size and metastasis. These 2 indicators, however, were often considered as related indicators to tumor progression or deterioration severity. Third, no significant difference was found between tissues with moderate and poor histological grade which cannot fully support our conclusion that RP11‐466I1.1 expression level was higher in HCC tissues with poorer histological grade. For these 2 problems, more samples should be recruited to validate our result.

Despite the limitations above, our study was the first attempt to investigate the correlation between RP11‐466I1.1 and cancers as it was discovered as an upregulated gene in response to LPS stimulation in HUVECS. The significant upregulation of RP11‐466I1.1 in HCC and its correlation with histological grade and tumor capsule integrity strongly supported that RP11‐466I1.1 may participate in HCC pathogenesis, especially the tumor cell proliferation and invasion.

However, the concrete mechanisms remain unknown. Given that lncRNAs play their roles by targeting related genes, the homology of RP11‐466I1.1 with LPA and PPAR‐gamma reminded us whether the 2 homologous genes may be involved in the acting mechanism of RP11‐466I1.1 in HCC. LPL is lipid metabolism‐related enzyme which can catalyze the hydrolysis of triglycerides to produce free fatty acids (FA) and increases the FA uptake by cells. A recent study reported by Cao et al^23^ found that LPL was upregulated in HCC tissues, and there is a correlation between the high expression of LPL and poor prognosis of HCC. On the other hand, PPAR‐gamma is a ligand‐inducible nuclear transcription factor, which has been demonstrated involving in carcinogenesis by regulating cell proliferation, differentiation, and survival through mediating the effects of fatty acids and their derivatives.^24^ Yu et al^25^ further reported the PPAR‐gamma expression changes in HCC. They found that expression levels of PPAR‐gamma were significantly downregulated in HCC tissues, and HCC with poor histological differentiation exhibited lower PPAR‐gamma expression level. Taken together, these findings suggest some potential correlations between RP11‐466I1.1 and these 2 homologous lipid metabolism‐related genes during the pathogenic progress of HCC, pointing the direction for further exploring the downstream regulating mechanisms of RP11‐466I1.1. And we deeply believe that further study on the function and mechanism of RP11‐466I1.1 will provide theoretical basis for improving the management of HCC.

In summary, our study found that the expression of RP11‐466I1.1 was upregulated in patients with HCC, both in tumor tissues and in serum. The further correlation analysis showed the high expression of RP11‐466I1.1 was correlated with poor prognosis of patients with HCC and may serve as a promising novel biomarker for the prognosis in HCC but not for diagnosis. The concrete mechanisms of RP11‐466I1.1 playing roles in HCC pathogenesis need further study. And bioinformatics analysis suggested us the potential correlations between RP11‐466I1.1 and the 2 lipid metabolism‐related genes, LPL and PPAR‐gamma, pointing out the direction for our following research.

## CONFLICT OF INTEREST

The authors have no conflict of interest.
